# Comprehensive analysis of passive generation of parabolic similaritons in tapered hydrogenated amorphous silicon photonic wires

**DOI:** 10.1038/s41598-017-03840-4

**Published:** 2017-06-19

**Authors:** Chao Mei, Feng Li, Jinhui Yuan, Zhe Kang, Xianting Zhang, Binbin Yan, Xinzhu Sang, Qiang Wu, Xian Zhou, Kangping Zhong, Liang Wang, Kuiru Wang, Chongxiu Yu, P. K. A. Wai

**Affiliations:** 1 State Key Laboratory of Information Photonics and Optical Communications, Beijing University of Posts and Telecommunications, P.O. Box72 (BUPT), 100876 Beijing, China; 2 Photonics Research Centre, Department of Electronic and Information Engineering, The Hong Kong Polytechnic University, Hung Hom, Kowloon Hong Kong; 3 Hong Kong Polytechnic University Shenzhen Research Institute, Shenzhen, 518057 China; 4 Department of Physics and Electrical Engineering, Northumbria University, Newcastle upon Tyne, NE1 8ST United Kingdom; 5 Department of Electronic Engineering, The Chinese University of Hong Kong, Shatin, NT Hong Kong

## Abstract

Parabolic pulses have important applications in both basic and applied sciences, such as high power optical amplification, optical communications, all-optical signal processing, etc. The generation of parabolic similaritons in tapered hydrogenated amorphous silicon photonic wires at telecom (*λ* ~ 1550 nm) and mid-IR (*λ* ≥ 2100 nm) wavelengths is demonstrated and analyzed. The self-similar theory of parabolic pulse generation in passive waveguides with increasing nonlinearity is presented. A generalized nonlinear Schrödinger equation is used to describe the coupled dynamics of optical field in the tapered hydrogenated amorphous silicon photonic wires with either decreasing dispersion or increasing nonlinearity. The impacts of length dependent higher-order effects, linear and nonlinear losses including two-photon absorption, and photon-generated free carriers, on the pulse evolutions are characterized. Numerical simulations show that initial Gaussian pulses will evolve into the parabolic pulses in the waveguide taper designed.

## Introduction

Parabolic pulses are desirable in many applications, e.g. high power optical amplification and ultrashort pulse generation^1–4^, highly coherent continuum for optical communications^5–7^, and all-optical signal processing^8–10^. The temporal profiles of most pulsed laser sources are Gaussian, Lorentzian or hyperbolic secant. Direct generation of parabolic pulses from a laser cavity is difficult. In the past decades, several schemes have been proposed to generate parabolic pulses from pulses with different profiles^5, 6, 8, 9, 11^. Among these different schemes, parabolic similaritons in amplifiers with normal dispersion^12–15^ or dispersion decreasing waveguides^16, 17^ showed distinct advantages.

Similaritons preserve their shapes during propagation. Classic chirp-free solitons in uniform anomalous dispersive media is the simplest example of similaritons. In media with slowly varying anomalous dispersion, solitons can still preserve the hyperbolic secant pulse shape while its pulse parameters evolve adiabatically to satisfy the soliton condition^18^. Self-similar propagation of chirped solitons is also found in short tapered media with varying anomalous dispersion^19^ or nonlinearity^20^. Normal dispersive media do not support soliton propagation, but chirped parabolic pulses can experience self-similar propagations in amplified or parameters varying media. Unlike the solitons in anomalous dispersion regime which are formed with a certain power threshold, the generation of parabolic similaritons is not sensitive to power, shape, and phase profile of the initial pulses. In normal dispersive fibers with constant gain coefficients, parabolic similaritons are asymptotic solutions of the systems^12^, which means a pulse propagating in such fibers will eventually evolve to a parabolic similariton. Generation of parabolic pulses based on asymptotic evolutions have been successfully demonstrated in rare-earth doped fiber amplifiers^21^, such as ytterbium-doped fiber amplifiers^12, 13^, erbium-doped fiber amplifiers^2, 14, 15^ and nonlinear amplifiers such as Raman amplifiers^22, 23^. The parabolic similaritons will not experience wave breaking^24^ which is the major limitation of high power optical pulses propagating in normal dispersive media. Furthermore, the nearly linear chirp of parabolic similaritons allows straightforward pulse compression of such pulses in dispersive media^25^. However, the use of amplifiers will increase the system complexity and inevitably introduce amplified spontaneous emission noise into the pulses. Besides the normal dispersive amplifiers, parabolic similaritons are also found in passive dispersion decreasing media in which the governing equation can be transformed to the equivalent form for normal dispersive amplifiers. The realization of an effective constant gain is the key of the generation of parabolic similaritons using passive parabolic pulse generation systems. In the last few years, passive parabolic pulse generation has been demonstrated in dispersion decreasing fibers^26–28^, comb-like dispersion decreasing fibers^29, 30^, and two-segment normally dispersive fibers^31–33^.

However, the above parabolic pulses generation schemes require kilometers long fibers because of the relative low nonlinearity of silica. Obviously, long silica fibers are not compatible with future on-chip systems. More important, it is impossible to fabricate such long waveguides on a single chip. Comparing to silica, silicon shows much higher nonlinearity. Efficient nonlinear generation of parabolic pulse has been demonstrated in silicon fibers^16^ and silicon photonic wires^17^. However, the unavoidable two-photon absorption (TPA) and free-carrier absorption (FCA) limit the application of silicon at the telecom band. Hydrogenated amorphous silicon (a-Si:H), which can be fabricated by back-end-of-line (BEOL) CMOS technology^34^, has emerged as a promising candidate because of its large nonlinear figure of merit (FOM). Here, FOM = *n*_2_/(*λ* × *β*_TPA_), where *n*_2_ is the nonlinear refractive index and *β*_TPA_ is the coefficient of TPA. The FOMs of several nonlinear materials are listed in Table 1 for comparison. In addition, the bandgap of a-Si:H (~1.6 eV) is larger than that of crystalline silicon (c-Si) (~1.16 eV)^35^, thus the TPA and FCA in a-Si:H are lower than that of c-Si at telecom wavelength^36, 37^. Tapered a-Si:H photonic wires (a-Si:H-PhWs) with subwavelength cross section size are ideally suitable for dispersion engineering similar to the c-Si^38, 39^. In particular, by changing the waveguide width, one can design a-Si:H-PhWs with normal (*β*_2_(*ω*) = *β* ^(2)^(*ω*) > 0) or anomalous (*β*_2_(*ω*) < 0) dispersions within the wavelength range of interest^40^. Moreover, the large intrinsic third-order nonlinearity of a-Si:H enables strong nonlinear pulse shaping of low power pulses within millimeter-long waveguides. To date, a-Si:H has not been used in parabolic pulse generation. Furthermore, in the proposed schemes of passive parabolic pulse generation, either in fibers or silicon waveguides, only the dispersion decreasing profile is investigated. We note that nonlinearity increasing fiber taper has been proposed to realize self-similar soliton compression. However, such nonlinearity engineering has not been investigated for parabolic pulse generation.

**Table 1:** Summary of FOMs for materials at 1550 nm and 2150 nm.

Wavelength	a-Si:H	a-Si:H	a-Si:H-N	c-Si	As_2_S_3_	As_2_Se_3_
1550 nm	1.62^37^	4.6^34^	5.68^41^	0.3 ~ 0.4^42^	10.4^44^	8^44^
2150 nm	29^37^	>4.6^34^	>5.68^41^	>1^43^	>10.4^44^	>8^44^

In this paper, we investigate theoretically the passive generation of parabolic similaritons in tapered a-Si:H waveguides. Although the dispersion decreasing scheme has also been discussed by other researchers, a novel nonlinearity increasing scheme in the normal dispersion regime based on self-similar theory is proposed and investigated for the first time. Besides, to elucidate the difference between these two schemes, dispersion decreasing waveguides (DDWs) and nonlinearity increasing waveguides (NIWs) are compared for parabolic pulse generation. The taper profiles for DDWs and NIWs are designed using the finite element characterization of the a-Si:H waveguides. The dynamics of parabolic pulse generation in both tapers are investigated by considering the higher-order effects and loss in the waveguide tapers. We then studied parabolic pulse generation at the telecom (*λ* ~ 1550 nm) and mid-IR (*λ* ≥ 2100 nm) wavelengths.

## Results

### Characterization and design of tapered a-Si:H-PhWs

**Figure 1:**
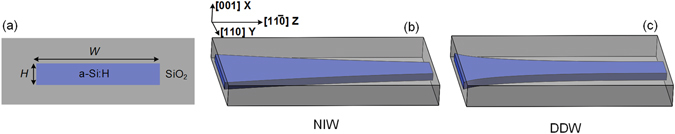
Schematics of the (**a**) cross-section, and the three-dimensional view of (**b**) nonlinearity increasing and (**c**) dispersion decreasing tapered a-Si:H-PhWs.

Figures 1(a–c) show the cross-section of the waveguide and the 3-dimensional sketches of the proposed NIWs and DDWs, respectively. Figure 1(a) shows the cross-section of the proposed tapered a-Si:H-PhWs which has a rectangular core buried in silica cladding. The height (*H*) of the waveguide is fixed at 220 nm. To engineer the nonlinearity or the dispersion of the waveguide by tapering the width (*W*), the waveguide with different *W* should be characterized first. We vary *W* from 500 to 3000 nm and calculate the corresponding propagation constants *β*(ω) of the fundamental quasi-TE mode in the spectral range of 1400 to 2400 nm. The waveguide is single mode within the whole spectral range. The complex nonlinear refractive index *n*_2_ of the material is obtained by polynomial fitting to the experimental data^37^. The polynomial fit is based on the theoretical predication of the dispersion of TPA and *n*_2_ of indirect semiconductors below the indirect bandgap^43, 45^. For each *W*, the dispersion coefficients *β*_2_ to *β*_10_ are obtained by the Taylor expansion of *β*(ω). Spline interpolation is used to increase the number of sampling points in the whole spectral range. Similar procedure is adopted to determine the frequency dependence of other waveguide parameters such as nonlinear coefficient *γ*(ω), self-steepening factor *τ*(ω), confinement factor *κ*(ω), and group velocity *v*_g_(ω).

**Figure 2:**
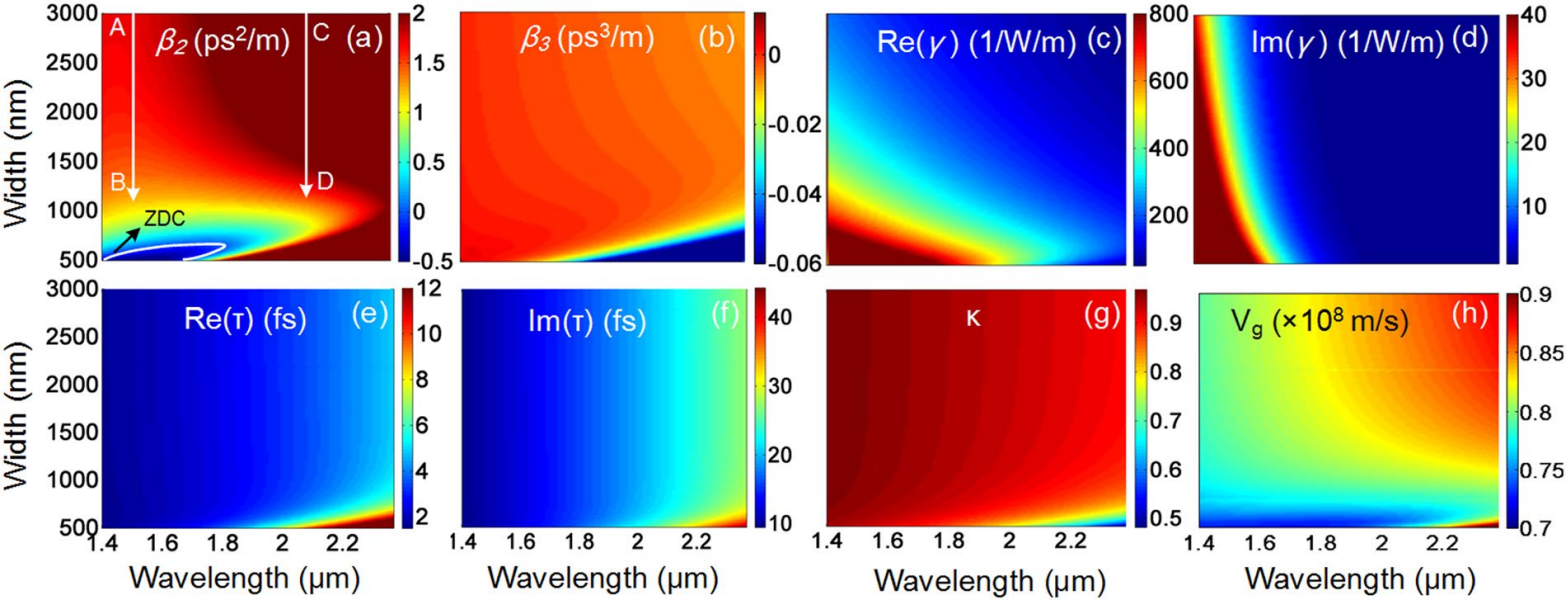
The variations of (**a**) *β*_2_, (**b**) *β*_3_, (**c**) real part of *γ*, (**d**) imaginary part of *γ*, (**e**) real part of *τ*, (**f**) imaginary part of *τ*, (**g**) confinement factor *κ*, and (**h**) group velocity *v*_g_versus the wavelength and taper width.

Figure 2 shows the variations of the linear and nonlinear optical coefficients of the proposed a-Si:H-PhWs versus waveguide width *W* and wavelength. The zero-dispersion curve (ZDC, white in color) in Fig. 2(a) shows that anomalous dispersion is confined to a small region with *λ* < 1690 nm and *W* < 680 nm, otherwise the waveguide will have normal dispersion. Thus, the proposed waveguide is particularly suitable for the generation of parabolic similaritons which requires normal dispersion. Figure 2(b) shows that the value of *β*_3_ is very low, thus third order dispersion will have little impact on the generation of parabolic similaritons. Figures 2(c,d) show the maps of the real (Re(*γ*)) and imaginary (Im(*γ*)) parts of *γ* respectively, which are small at 2150 nm. Figures  2(e,f) depict the real part (Re(*τ*)) and imaginary part (Im(*τ*)) of *τ*, respectively. Figures 2(g,h) show the dispersion of confinement factor *к* and group velocity *v*_g_, respectively.

**Figure 3:**
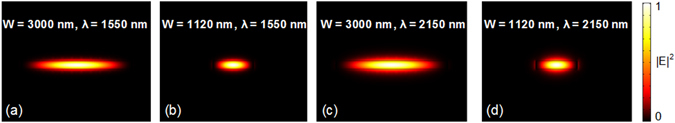
Optical field distributions at (**a**) input port with *W* = 3000 nm and *λ* = 1550 nm, (**b**) output port with *W* = 1120 nm and *λ* = 1550 nm, (**c**) input port with *W* = 3000 nm and *λ* = 2150 nm, and (**d**) output port with *W* = 1120 nm and *λ* = 2150 nm.

From Fig. 2(a), the normal dispersion coefficients at both the telecom wavelength 1550 nm and mid-IR wavelength 2150 nm decrease when *W* decreases. For comparison, we choose the same initial *W* = 3000 nm and the same final *W* = 1120 nm for all of the waveguide tapers investigated in this paper. The arrows AB and CD in Fig. 2(a) show the dispersion decreasing traces at 1550 nm and 2150 nm, respectively. The group velocity dispersion (GVD) and *γ* at the input and output ports are: (i) at 1550 nm, *β*_2,in_ = 1.76 ps^2^/m, *β*_2,out_ = 1.21 ps^2^/m, *γ*_in_ = 175.17/W/m, and *γ*_out_ = 481.85/W/m, and (ii) at 2150 nm, *β*_2,in_ = 2.83 ps^2^/m, *β*_2,out_ = 1.53 ps^2^/m, *γ*_in_ = 70.46/W/m, and *γ*_out_ = 196.68/W/m. Tables 2 and 3 show the higher-order dispersion coefficients calculated. The optical field distributions obtained by finite element method, as shown in Figs. 3(a–d)

**Table 2:** *β*_n_ at 1550 nm.

Width	*β*_2_ (ps^2^/m)	*β*_3_ (ps^3^/m)	*β*_4_ (ps^4^/m)	*β*_5_ (ps^5^/m)	*β*_6_ (ps^6^/m)	*β*_7_ (ps^7^/m)	*β*_8_ (ps^8^/m)	*β*_9_ (ps^9^/m)	*β*_10_(ps^10^/m)
3 μm	1.76	−1.11 × 10^−3^	1.04 × 10^−5^	5.2 × 10^−9^	4.33 × 10^−10^	5.83 × 10^−12^	8.55 × 10^−14^	8.73 × 10^−16^	4.15 × 10^−18^
0.5 μm	−0.619	−2.59 × 10^−4^	8.81 × 10^−5^	−1.81 × 10^−6^	−2.63 × 10^−8^	3.44 × 10^−10^	4.86 × 10^−11^	8.81 × 10^−13^	4.48 × 10^−15^

**Table 3:** *β*_n_ at 2150 nm.

Width	*β*_2_ (ps^2^/m)	*β*_3_ (ps^3^/m)	*β*_4_ (ps^4^/m)	*β*_5_ (ps^5^/m)	*β*_6_ (ps^6^/m)	*β*_7_ (ps^7^/m)	*β*_8_(ps^8^/m)	*β*_9_ (ps^9^/m)	*β*_10_ (ps^10^/m)
3 μm	2.83	−5.71 × 10^−3^	1.89 × 10^−5^	−3.66 × 10^−8^	−1.12 × 10^−11^	2.68 × 10^−14^	2.83 × 10^−14^	−5.35 × 10^−16^	3.1 × 10^−18^
0.5 μm	20.8	−0.188	2.82 × 10^−4^	2.89 × 10^−5^	−6.04 × 10^−7^	5.38 × 10^−9^	7.63 × 10^−12^	−6.39 × 10^−13^	3.8 × 10^−15^

, indicate that the light fields are well confined in the core region of the a-Si:H-PhWs during the whole propagation at both 1550 nm and 2150 nm.

**Figure 4:**
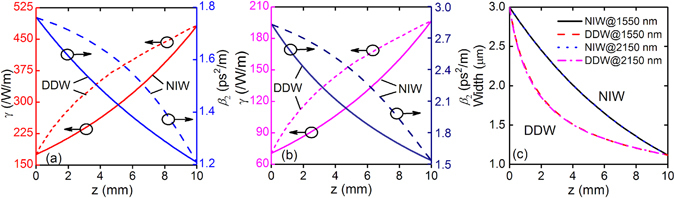
Variations of nonlinear coefficient *γ* and dispersion *β*_2_ in NIWs and DDWs with pump wavelengths at (**a**) 1550 nm and (**b**) 2150 nm. The solid curves are the designed parameters and the dashed curves are the corresponding parameters. (**c**) The taper width profile *W*(*z*) of NIWs and DDWs with pump wavelengths 1550 nm and 2150 nm.

In order to generate parabolic pulses in the tapered waveguides, the taper profile should be carefully designed to support self-similar propagation of parabolic similaritons. It is known that asymptotic parabolic similariton solutions can be found in a normal dispersive medium with a dispersion decreasing profile with *β*_2_ = *β*_20_/(1 + *b*_0_z) and constant nonlinearity, as described by Eqs. (15–23). In this paper, we showed that such asymptotic parabolic similariton solutions can also be found in a nonlinearity increasing medium with *γ* = *γ*_0_exp(*a*_0_ *z*) and constant dispersion *β*_2_, as derived in Eqs. (7–14). In the following, we classify the tapered waveguides with *γ* = *γ*_0_exp(*a*_0_ *z*) as NIWs and that with *β*_2_ = *β*_20_/(1 + *b*_0_ *z*) as DDWs based on the governing equations in the ideal cases i.e. (7) and (15), respectively, despite possible variations of *β*_2_ in NIWs and *γ* in DDWs. When the length of the taper is chosen and the conditions defined by points A, B, C and D in Fig. 2(a) are applied to Eqs. (9) and (19), the taper parameters *a*_0_ in Eq. (9) and *b*_0_ in Eq. (19) will be determined. In our examples, the taper length is fixed at 10 mm. The designed profiles of the tapered a-Si:H-PhWs are (i) *γ* = *γ*_0_exp(*a*_0_ *z*) for the NIW at 1550 nm, where *γ*_0_ = 175.17/W/m and *a*_0_ = 101.19 /m, (ii) *γ* = *γ*_0_exp(*a*_0_ *z*) for the NIW at 2150 nm, where *γ*_0_ = 70.46/W/m and *a*_0_ = 102.66 /m, (iii) *β*_2_ = *β*_20_/(1 + *b*_0_ *z*) for the DDW at 1550 nm, where *β*_20_ = 1.76 ps^2^/m and *b*_0_ = 45.4 /m, and (iv) *β*_2_ = *β*_20_/(1 + *b*_0_ *z*) for the DDW at 2150 nm, where *β*_20_ = 2.83 ps^2^/m and *b*_0_ = 84.5 /m. Figures 4(a,b) respectively show the variations of the dispersion and nonlinearity (solid curves) along the taper length at 1550 nm and 2150 nm, respectively. For a given dispersion or nonlinearity curve versus *z* and by using the data in Fig. 2, the taper profiles of NIWs and DDWs can be obtained, as shown in Fig. 4(c). We observed that the curvatures of the taper profiles of DDWs are much larger than that of NIWs. The taper profiles of NIWs are quasi-linear but that of DDWs are exponential-like. The taper profiles at 1550 nm and 2150 nm are almost same. Thus it is feasible to fabricate a single taper that can be used for both the telecom and mid-IR bands. Figures 1(b,c) show the corresponding three-dimensional schematics of the NIWs and DDWs, respectively. We emphasize that although the tapers NIWs and DDWs are designed according to their corresponding nonlinearity and dispersion profiles, the nonlinearity or dispersion cannot be engineered individually without affecting the other. With the taper profiles in Fig. 4(c), the corresponding variation of dispersion in NIWs and that of nonlinearity in DDWs can be obtained, as shown by the dashed curves in Figs. 4(a,b). In NIWs, the dispersion decreases along the taper length but the profile of decrease is different from that of DDWs. Similarly, nonlinearity increasing curves are observed in DDWs. Since in an ideal NIW (DDW), the dispersion (nonlinearity) should remain constant in the whole taper, the variation of dispersion (nonlinearity) will introduce deviations from the ideal case in propagation. We observed that the waveguides have higher nonlinearity and lower dispersion at 1550 nm than that at 2150 nm.

### Parabolic similariton generation in ideal tapers

Before the full simulations with all the parameters shown in Fig. 4, it is worthy to consider the ideal cases where only the nonlinearity or dispersion is varied in NIWs or DDWs, respectively. From the self-similar theory, Eq. (7) requires a constant *β*_2_ for NIWs, and Eq. (15) requires a constant *γ* for DDWs, respectively. In realistic tapers, the *β*_2_ of NIWs and *γ* of DDWs vary with taper lengths. Thus, we adopt the effective *β*_2_ and *γ* which are obtained by averaging the nonlinearity and dispersion coefficient along the taper length. The effective *β*_2_ and *γ* are defined as

1a $${\beta }_{2,eff}=\frac{1}{L}{\int }_{0}^{L}{\beta }_{2}(z)dz,$$

1b $${\gamma }_{eff}=\frac{1}{L}{\int }_{0}^{L}\gamma (z)dz.$$

where *β*_2,*eff*_ and *γ*_*eff*_ are 1.56 ps^2^/m and 363.63/W/m at 1550 nm, 2.32 ps^2^/m and 148.48/W/m at 2150 nm, respectively.

**Figure 5:**
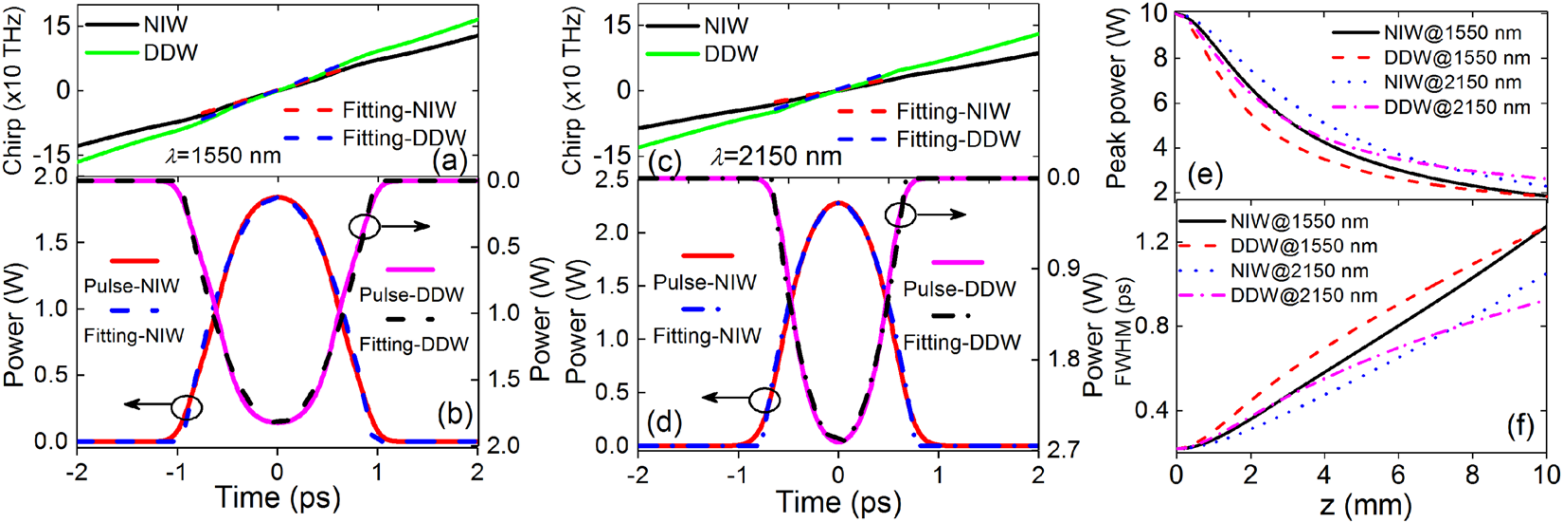
(**a**) Chirp profiles (solid curves) and their linear fits (dashed curves) for NIW (black and red curves) and DDW (green and blue curves) at 1550 nm. (**b**) Output pulse waveforms (solid curves) and their parabolic fits (dashed curves) for NIW (red and magenta curves) and DDW (blue and black curves) at 1550 nm. (**c**) Chirp profiles (solid curves) and their linear fits (dashed curves) for NIW (black and red curves) and DDW (green and blue curves) at 2150 nm. (**d**) Output pulse waveforms (solid curves) and their parabolic fits (dash-dotted curves) for NIW (red and blue curves) and DDW (magenta and black curves) at 2150 nm. (**e**) The peak power of propagating pulses for NIW (black solid curves) and DDW (red dashed curves) at 1550 nm, and NIW (blue dotted curves) and DDW (magenta dash-dotted curves) at 2150 nm. (**f**) The FWHM of propagating pulses, for NIW (black solid curves) and DDW (red dashed curves) at 1550 nm, and NIW (blue dotted curves) and DDW (magenta dash-dotted curves) at 2150 nm.

A Gaussian pulse with amplitude *A*(*t*) = *A*_0_ exp(−*t* ^2^/2${t}_{0}^{2}$), where the full-width-half-maximum (FWHM) duration *t*_FWHM_ = 1.665*t*_0_ = 220 fs, is launched into the tapered a-Si:H-PhWs. We note that the parabolic similariton is partly determined by the profile of initial pulses. A super-Gaussian pulse can evolve into a similariton over a shorter distance^17^. However, for simplicity, we will not discuss the impact of initial pulse shape on the generation of parabolic similariton in this work. The initial pulse profile is chosen to be Gaussian. The peak power of the initial pulse, ${P}_{0}={A}_{0}^{2}$ is 10 W. The input pulse energy is 2.34 pJ calculated by using the formula (π/ln2)^1/2^ *t*_FWHM_ *P*_0_/2. The pulse propagation is modeled by Eq. (7) for NIWs and Eq. (15) for DDWs. Figures 5(a–d) show the chirp profiles and waveforms of the output pulses at 1550 and 2150 nm, respectively. In Figs. 5(a,c), both the pulses at 1550 and 2150 nm show positive linear chirp. The waveforms in Figs. 5(b,d) are fitted by a parabolic function $|{u}_{p}(t){|}^{2}=|{u}_{p}({t}_{0}){|}^{2}[1-{(t-{t}_{0})}^{2}/{T}_{p}^{2}]$ for $|t-{t}_{0}| < {T}_{p}$ and ${u}_{p}(t)=0$ otherwise, where *t*_0_ is the position of the pulse and *T*_*p*_ is the pulse width. The fitting results confirm the parabolic profiles of the central part of the pulses. The results indicate that the input Gaussian pulses have evolved into parabolic pulses which are the asymptotic solutions as described by the self-similar theory. Figure 5(e) shows the rapid drop in the pulse peak powers during propagation in different tapers and at different wavelengths. Figure 5(f) shows that the FWHMs of the pulse increase monotonically along *z*. The results qualitatively agree with the prediction of Eqs. (11–14) and (20–23). However, we note that the effective parameters do not fully describe the corresponding variations of *β*_2_ and *γ* in tapered a-Si:H-PhWs NIWs and DDWs, respectively. Furthermore, higher-order effects (HOEs) including higher-order dispersions and nonlinear effects, linear and nonlinear losses have not been included. Thus, the model of Eq. (2) should be adopted in the investigation of realistic cases.

### Parabolic similariton generation in tapered a-Si:H-PhWs with higher-order effects and losses

In realistic tapered waveguides, the physical effects are more complex than that described by Eqs. (7) and (15). Optical pulse evolution in tapered a-Si:H-PhWs can be described by the generalized nonlinear Schrödinger equation (GNLSE)^46–48^

2 $$\begin{array}{c}i\frac{{\rm{\partial }}A}{{\rm{\partial }}z}+\sum _{n=2}^{10}\frac{{i}^{n}{\beta }_{n}(z)}{n!}\frac{{{\rm{\partial }}}^{n}A}{{\rm{\partial }}{t}^{n}}+\frac{ic\kappa (z)}{2{n}_{0}{v}_{g}(z)}({\alpha }_{{\rm{F}}{\rm{C}}}+{\alpha }_{l})A=-\frac{\omega \kappa (z)}{{n}_{0}{v}_{g}(z)}\delta {n}_{{\rm{F}}{\rm{C}}}(z)A\\ \,\,\,\,\,\,\,\,\,\,\,\,\,\,\,\,\,\,\,\,\,\,\,\,\,\,\,\,\,\,\,\,\,\,\,\,\,\,\,\,\,\,\,\,\,\,\,\,\,\,\,\,\,\,\,\,\,\,\,\,\,\,\,\,\,\,\,\,\,\,\,\,\,\,\,\,\,\,\,\,\,\,\,\,\,\,\,\,\,\,\,\,\,\,\,\,\,\,\,\,\,\,\,\,\,\,\,\,\,\,\,\,\,-\gamma (z)[1+i\tau (z)\frac{{\rm{\partial }}}{{\rm{\partial }}t}]{|A|}^{2}A,\end{array}$$

where *A*(*z*, *t*) is the slowly varying envelope of the pulse, *z* and *t* are the propagation distance and time in the frame co-moving with the group velocity *v*_*g*_(*z*) of the optical pulse, respectively. *β*_*n*_(*z*) = *d* ^*n*^ *β*/*d*ω^*n*^ is the *n-*th order dispersion coefficient. *κ*(*z*) is the confinement factor of the guiding layer. *c* and *n*_0_ are the speed of light in vacuum and refractive index of a-Si:H-PhWs, respectively. *α*_FC_ and *α*_*l*_ are the FCA and linear losses, which are ~2 dB/cm at 1550 nm and ~0.5 dB/cm at 2150 nm^37^. *δn*_FC_ and *τ* are the free-carrier induced refractive index change and the self-steepening factor. *α*_FC_ and *δn*_FC_ are given by^46–48^.

3 $${\alpha }_{{\rm{F}}{\rm{C}}}(z)=\frac{{e}^{3}N(z)}{{\varepsilon }_{0}c{n}_{0}{\omega }^{2}}(\frac{1}{{\mu }_{{\rm{e}}}{m}_{{\rm{c}}{\rm{e}}}^{2}}+\frac{1}{{\mu }_{{\rm{h}}}{m}_{{\rm{c}}{\rm{h}}}^{2}}),$$

4 $${\delta n}_{{\rm{F}}{\rm{C}}}(z)=\frac{-{e}^{2}}{2{\varepsilon }_{0}{n}_{0}{\omega }^{2}}(\frac{N(z)}{{m}_{{\rm{c}}{\rm{e}}}}+\frac{N{(z)}^{0.8}}{{m}_{{\rm{c}}{\rm{h}}}}),$$

where *N* is the free carrier density, *m*_ce_ = 0.5*m*_0_ (*m*_ch_ = *m*_0_, *m*_0_ is the electron mass) is the electron (hole) effective mass, and *μ*_e_(*μ*_h_) is the electron (hole) mobility. The complex nonlinear coefficient *γ*(*z*) = *γ*′(*z*) + *iγ*″(*z*) = (*n*_2_ *ω* + *icβ*_TPA_/2)×*n* ^2^ *c*[*A*_0_(*z*)*v*_g_^2^(*z*)]^−1^, where *n*_2_ and *β*_TPA_ are the nonlinear refractive index and TPA coefficient of a-Si:H, respectively. The TPA coefficients (*β*_TPA_) are 3.69 × 10^−14 ^m/W at 2150 nm and 6.95 × 10^−12 ^m/W at 1550 nm^37^, respectively. The shock time *τ*(*z*) = ∂ln*γ*(*z*)/∂ω. *A*_0_(*z*) is the transverse dimension of the waveguide. The rate equation of *N*(*z*) is given by^46–48^

5 $$\frac{{\rm{\partial }}N}{{\rm{\partial }}t}=-\frac{N}{{t}_{{\rm{c}}}}+\frac{{\gamma }^{{\rm{^{\prime} }}{\rm{^{\prime} }}}}{\hslash \omega {A}_{0}(z)}{|A|}^{4},$$

where *t*_c_ is the free-carrier lifetime set at 400 ps in the simulations.

Equations (2) and (5) describe optical pulse propagation in an a-Si:H-PhWs with varying transverse dimension since the waveguide parameters are incorporated in our model via the implicit dependence of the a-Si:H-PhWs modes on the transverse dimension. The intra-pulse Raman scattering in a-Si:H-PhWs is negligible and is not considered here^37, 38^. The input pulse is Gaussian, the same as that used in the ideal case.

**Figure 6:**
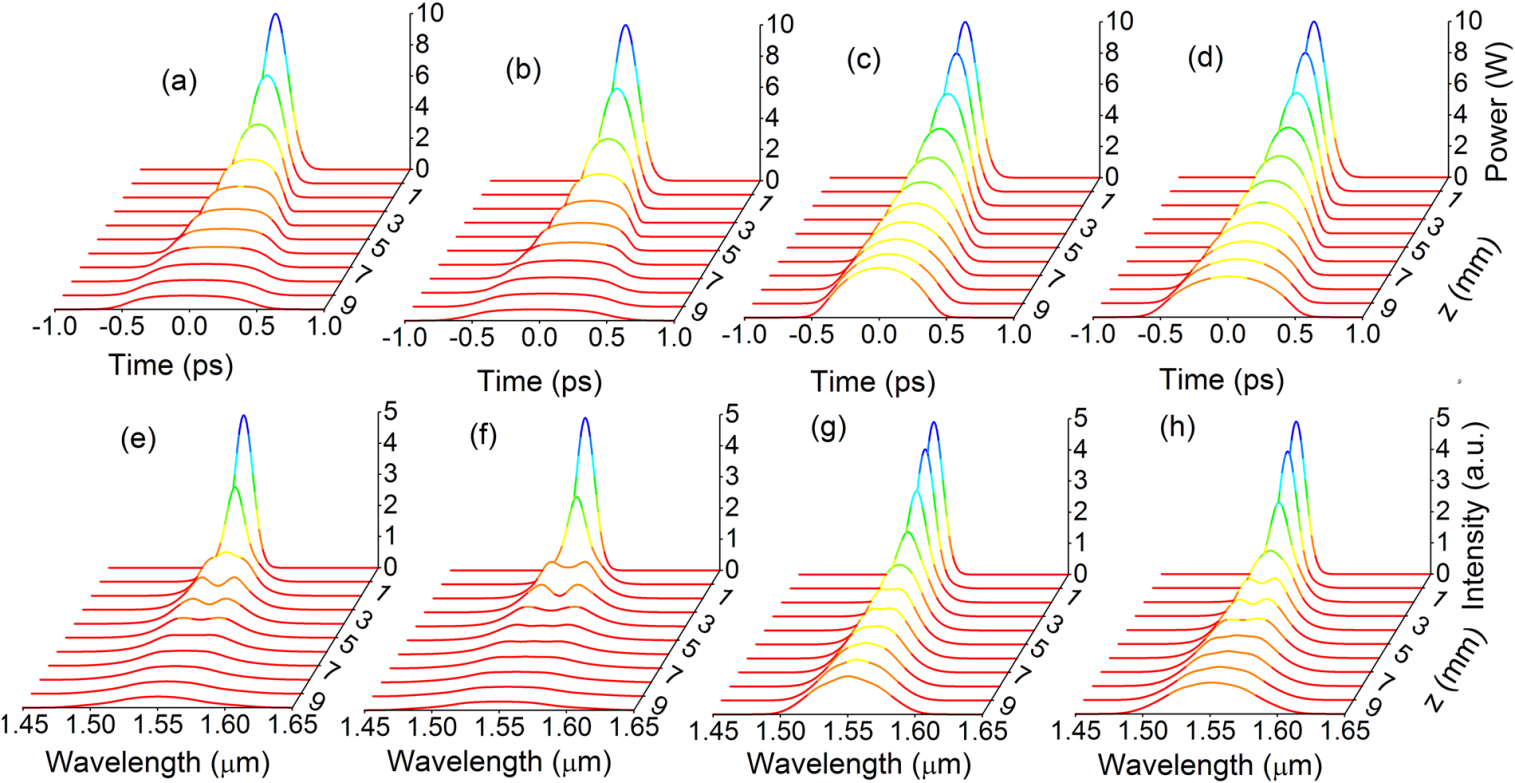
Evolutions of the Gaussian input pulses along the taper modeled by the GNLSE. The evolutions of the waveforms in the top row are obtained in (**a**) NIWs at 1550 nm, (**b**) DDWs at 1550 nm, (**c**) NIWs at 2150 nm and (**d**) DDWs at 2150 nm. The evolutions of the spectra in the bottom row are obtained in (**e**) NIWs at 1550 nm, (**f**) DDWs at 1550 nm, (**g**) NIWs at 2150 nm and (**h**) DDWs at 2150 nm.

Figure 6 shows the evolutions of temporal waveforms and spectra of the pulses in NIWs and DDWs with pump wavelengths at 1550 and 2150 nm. Figures 6(a–d) show the temporal waveforms and Figs. 6(e–h) show the corresponding optical spectra. The left two columns show the evolutions in NIW and DDW pumped at 1550 nm. The right two columns show the evolutions in NIW and DDW pumped at 2150 nm. The peak pulse powers in Figs. 6(a,b) are much lower than that in Figs. 6(c,d), which indicates that the losses at 1550 nm are much higher than that at 2150 nm. The output pulses at 1550 nm are also longer and flatter than that at 2150 nm. In the spectral domain, the higher nonlinearity at 1550 nm generates much higher sidebands than that at 2150 nm, which will introduce larger perturbation to the asymptotic convergence to the parabolic similaritons. Comparing the pulse evolutions in the two different waveguides at *z* = 10 mm, we observed that the peak powers with NIWs in Figs. 6(a,c) are slightly higher than that with DDWs in Figs. 6(b,d), respectively. The difference between the pulse evolutions in NIWs and DDWs is due to the different contribution of the different varying *β*_2_ and *γ*. We also observed that for NIWs the spectrum develops twin peaks, a signature of the SPM induced nonlinear phase change, at *z* ~ 3 mm (Fig. 6(e)), while for DDWs the twin peak spectral feature occurs at *z* ~ 2 mm (Fig. 6(f)). Thus the nonlinear phase change induced by SPM in DDW is stronger that in NIW. We observed similar effects in Figs. 6(g,h).

To compare the parabolic similaritons generated in different tapers with different parameters, we defined a mismatch factor *δ* as^28^

6 $${\delta }^{2}=\frac{\int {[P(t)-{P}_{f}(t)]}^{2}dt}{\int {P}^{2}(t)dt},$$

where *P*(*t*) is the power of the generated similaritons and *P*_*f*_ is the power of the fitting pulse. The value of *δ* ^2^ will indicate how close the generated pulse resembles a parabolic pulse. A zero *δ* ^2^ means an ideal parabolic pulse. We will study the evolutions of the pulses at 1550 nm and 2150 nm in both NIWs and DDWs in the following.

**Figure 7:**
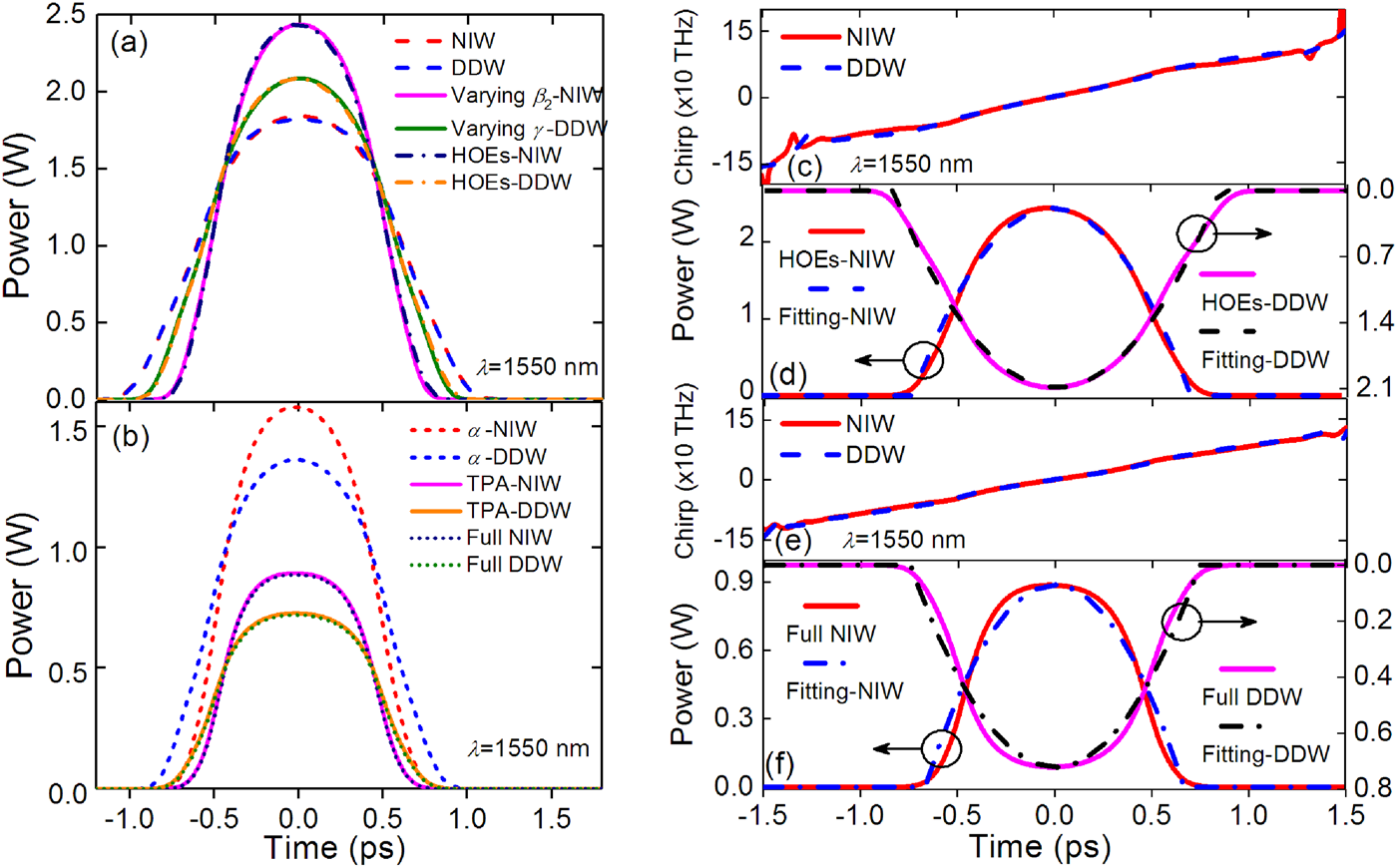
Output pulses with pump wavelength 1550 nm. (**a**) Output pulse waveforms with ideal NIW (red dashed curve), ideal DDW(blue dashed curve), varying *β*_2_-NIW (magenta solid curve), varying *γ*-DDW (orange solid curve), HOEs-NIW (navy dash-dotted curve), and HOEs-DDW (olive dash-dotted curve), (**b**) Output pulse waveforms with lossy terms in *α*-NIW (red short dashed curve), *α*-DDW (blue short dashed curve), TPA-NIW (magenta solid curve), TPA-DDW (orange solid curve), Full NIW (navy dotted curve) and Full DDW (olive dotted curve). (**c**) Chirp profiles of pulses from HOEs-NIW (red solid curves) and HOEs-DDW (blue dashed curve). (**d**) Output pulses of HOEs-NIW (red solid curve), parabolic fit for NIW (blue dashed curve), HOEs-DDW (magenta solid curve), and parabolic fit for DDW (black dashed curve). (**e**) Chirp profiles of pulses from Full NIW (red solid curve) and Full DDW (blue dashed curve). (**f**) Output pulses of Full NIW (red solid curve), parabolic fit for NIW (blue dash-dotted curve), Full DDW (magenta solid curve), and parabolic fit for DDW (black dash-dotted curve).

Figure 7 shows the output pulses at 1550 nm from NIW and DDW. To investigate the impact of each effect, the varying *β*_2_ or *γ*, HOEs (higher-order dispersions and self-steepening), linear loss *α*, TPA, FCA and FCD are added into the GNLSE model one by one, which correspond to the curves with prefixes “varying *β*_2_- or *γ*-”, “HOEs-”, “*α*-”, “TPA-”, and “Full”, respectively. The full model included all of the effects above.

In Fig. 7(a), the pulse peak power of the varying *β*_2_-NIW is 2.44 W, which is larger than the peak power 1.84 W of ideal NIW. Clearly, the decreasing *β*_2_ of the NIW has weakened the linear pulse stretching induced by dispersion. From Fig. 6, the spectrum of the input pulse is broadened by the nonlinearity. The mismatch between the strength of dispersion and the nonlinearity lead to a narrower pulse width in varying *β*_2_-NIW when compared to the ideal NIW. Similar impact has been observed in DDW when the varying *γ* is included. In Fig. 7(a), the HOEs are found to play minor role in the similariton generation and the pulses generated are almost the same as those generated in varying *β*_2_-NIWs and varying *γ*-DDWs except a slight asymmetry on the waveforms. The results are expected because *β*_3_ and *τ* at the operation wavelength are very small. In contrast, the losses *α* and TPA significantly affect the propagation. From Fig. 7(b), the pulse peak power of *α*-NIW (1.58 W) and TPA-NIW (0.89 W) are much lower than that of HOEs-NIW (2.44 W), and the pulse peak power of *α*-DDW (1.36 W) and TPA-DDW (0.73 W) are much lower than that of HOEs-DDW (2.09 W). Clearly, the linear and nonlinear losses attenuated the pulse and weakened the nonlinearity in the propagation. Finally, we observe that the FCA and FCD have almost no influence on the pulse evolutions since the “Full NIW” and “Full DDW” curves overlap well with their corresponding “TPA-” curves.

To estimate the quality of the parabolic pulses shown in Figs. 7(a,b), the chirp and temporal profiles of the output pulses from “HOEs-” and “Full” models are shown in Figs. 7(c–f). From Fig. 7(d), the generated pulses agree well with their parabolic fits. In Fig. 7(f), the generated pulses profiles with the full model deviate more significantly from the parabolic fits than those in Fig. 7(d). We note that the losses, especially the TPA, significantly degrade the generation of parabolic similariton. Figures 7(c,e) show that the chirp profiles are almost linear, especially at the center part of the pulses.

**Figure 8:**
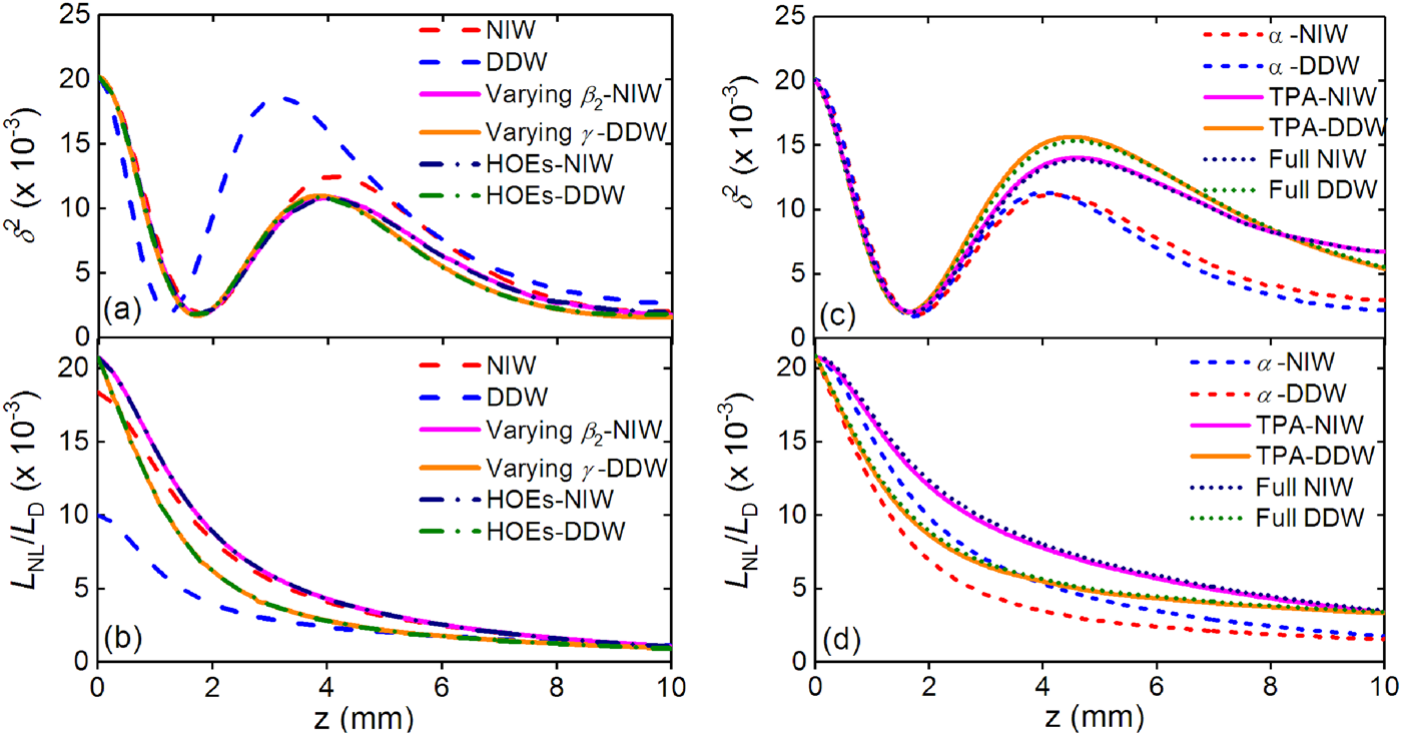
(**a**) The mismatch factor *δ* ^2^ and (**b**) ratio *L*_NL_/*L*_D_ at 1550 nm for NIW (red dashed curves), DDW (blue dashed curves), varying *β*_2_-NIW (magenta solid curves), varying *γ*-DDW (orange solid curves), HOEs-NIW (navy dash-dotted curves) and HOEs-DDW (olive dash-dotted curves). (**c**) The mismatch factor *δ* ^2^ and (**d**) ratio *L*_NL_/*L*_D_ at 1550 nm for *α*-NIW (blue dashed curves), *α*-DDW (red dashed curves), TPA-NIW (magenta solid curves), TPA-DDW (orange solid curves), Full NIW (navy dash-dotted curves), and Full DDW (olive dash-dotted curves).

Figures 8(a,c) show the evolutions of *δ* ^2^ along the propagation described by the models corresponding to those in Figs. 7(a,b). We find that all the *δ* ^2^ curves go through damped oscillations indicating that the input pulses converge to the parabolic similaritons. Figure 8(a) shows the variations of *δ* ^2^ along the propagation without losses. The *δ* ^2^ of DDW reaches its minimum value of 1.96 × 10^−3^ at *z* = 1.2 mm, and other curves reach their minimum values at *z* ~ 4 mm. However, the *δ* ^2^ of DDW is larger than that of all other models in most of the propagation. Figure 8(a) shows that the inclusions of the varying *β*_2_ (for NIWs) and *γ* (for DDWs) change the evolution curves significantly, but the deviations caused by the inclusion of HOEs are negligible, which agrees with the results in Fig. 7(a). In Fig. 8(b), the ratio of the nonlinear length *L*_NL_ = 1/(*γP*) and dispersion length *L*_D_ = *t* ^2^/*β*_2_ are investigated to estimate the relative contribution of nonlinearity in the propagation. As expected, the ratio *L*_NL_/*L*_D_ of DDW in Fig. 8(b) is much lower than that of other models before *z* = 5 mm, which indicates that DDWs have the strongest nonlinearity in Fig. 8(a). Although the evolution curves of *δ* ^2^ and *L*_NL_/*L*_D_ in Figs. 8(a,b) are different, they all approach to comparable values at the end of propagation.

When losses are included, all the curves in Fig. 8(c) reach approximately the same minimum value of 2.07 × 10^−3^ at *z* ~ 1.71 mm. At the output port of the waveguides, the *δ* ^2^ value for Full DDW is 5.51 × 10^−3^ which is higher than the 1.82 × 10^−3^ for HOEs-DDW. Similarly, *δ* ^2^ increases from 2.02 × 10^−3^ for HOEs-NIW to 6.78 × 10^−3^ for Full NIW. The increases of *δ* ^2^ are caused by losses. We note that the variation of *δ* ^2^ of Full NIW and Full DDW overlap in the region of 0 < *z* < 1.73 mm, but deviate from each other after the minimum points. Figure 8(d) shows that the inclusion of TPA weakens the Kerr nonlinearity and degrades the quality of the generated pulses further because of the shaping effect from the different losses to different parts of the pulse. Thus losses, especially the nonlinear loss, play a more important role than the waveguide structure in evolution of the initial Gaussian pulses into parabolic similaritons.

From Figs. 7 and 8, parabolic pulses have been successfully obtained in both NIW and DDW tapers. The input Gaussian pulse will asymptotically converge to parabolic similaritons. The HOEs and free carrier related effects have only minor influence to the pulse evolutions. The varying *β*_2_ in NIWs and *γ* in DDWs weaken the pulse stretching and lead to shorter and higher power pulses than the ideal cases. The losses in a-Si:H, especially the TPA, are the dominant effects that attenuate and degrade the quality of the generated parabolic pulses. From Fig. 2, the a-Si:H-PhWs have much lower loss at 2150 nm than 1550 nm. The TPA coefficient *β*_TPA_ = 3.69 × 10^−14^ m/W at 2150 nm is much lower than the value 6.95 × 10^−12^ m/W at 1550 nm. Thus the degradation caused by losses is expected to be greatly mitigated if the pump wavelength is changed to 2150 nm.

**Figure 9:**
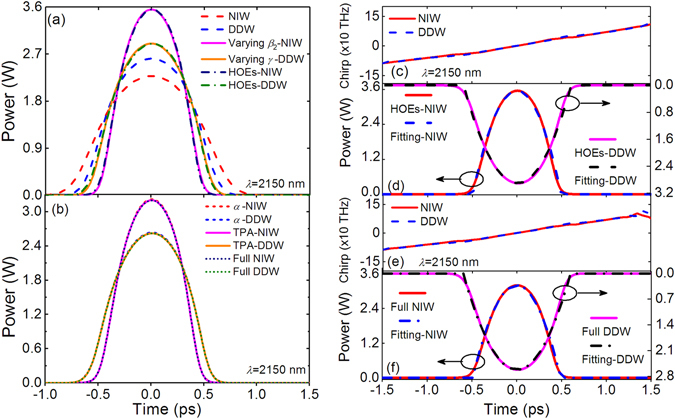
Output pulses with pump wavelength 2150 nm. (**a**) Output pulse waveforms with ideal NIW (red dashed curve), ideal DDW (blue dashed curve), varying *β*_2_-NIW (magenta solid curve), varying *γ*-DDW (orange solid curve), HOEs-NIW (navy dash-dotted curve), and HOEs-DDW (olive dot-dashed curve), (**b**) Output pulse waveforms with lossy terms in *α*-NIW (red short dashed curve), *α*-DDW (blue short dashed curve), TPA-NIW (magenta solid curve), TPA-DDW (orange solid curve), Full NIW (navy dotted curve) and Full DDW (olive dotted curve). (**c**) Chirp profiles of pulses from HOEs-NIW (red solid curves) and HOEs-DDW (blue dashed curve). (**d**) Output pulses of HOEs-NIW (red solid curve), parabolic fit for NIW (blue dashed curve), HOEs-DDW (magenta solid curve), and parabolic fit for DDW (black dashed curve). (**e**) Chirp profiles of pulses from Full NIW (red solid curve) and Full DDW (blue dashed curve). (**f**) Output pulses of Full NIW (red solid curve), parabolic fit for NIW (blue dot-dashed curve), Full DDW (magenta solid curve), and parabolic fit for DDW (black dot-dashed curve).

Figure 9 shows the parabolic pulses generated and their chirp profiles with pump pulses at 2150 nm. Figures 9(a,b) show the output pulses from models including different effects. Figure 9(a) shows that the output pulse from the ideal NIW has a lower peak power and longer pulse width than that from ideal DDW at 2150 nm. This is different from that at 1550 nm, where the output pulses from ideal NIW and ideal DDW are almost same, as shown in Fig. 7(a). But when other effects are included, e.g. in the full model, the pulse from NIW is higher in peak power and shorter in pulse width than that from DDW, which agree with the results at 1550 nm. The impacts of HOEs and the varying *β*_2_ (for NIWs) and *γ* (for DDWs) are similar to that observed at 1550 nm in Fig. 7(a). Figure 9(b) shows that the major difference between the different pump wavelengths is the impact of the losses. To be more specific, the peak power of output pulse at 2150 nm drops by 9.6% from 3.55 W (HOEs-NIWs) to 3.21 W (*α*-NIWs), which is much smaller than the 35% drop at 1550 nm. When the TPA, FCA and FCD are also included, the peak power only decreases slightly by 0.01 W to 3.2 W which indicates that the nonlinear losses are very low at 2150 nm. More importantly, the generated pulses agree well with the parabolic fit curves in both Figs. 9(d,f). Thus impact of losses at 2150 nm is much smaller than that at 1550 nm.

**Figure 10:**
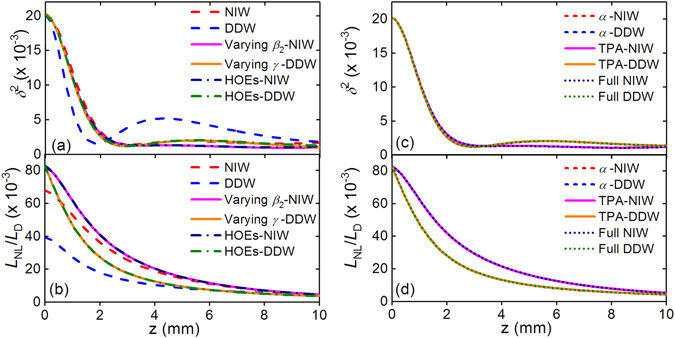
(**a**) The mismatch factor *δ* ^2^ and (**b**) ratio *L*_NL_/*L*_D_ at 2150 nm for NIW (red dashed curves), DDW (blue dashed curves), varying *β*_2_-NIW (magenta solid curves), varying *γ*-DDW (orange solid curves), HOEs-NIW (navy dot-dashed curves) and HOEs-DDW (olive dot-dashed curves). (**c**) The mismatch factor *δ* ^2^ and (**d**) ratio *L*_NL_/*L*_D_ at 2150 nm for *α*-NIW (blue dashed curves), *α*-DDW (red dashed curves), TPA-NIW (magenta solid curves), TPA-DDW (orange solid curves), Full NIW (navy dot-dashed curves), Full DDW (olive dot-dashed curves).

Figure 10 shows the evolutions of *δ* ^2^ and *L*_NL_/*L*_D_ for the parabolic pulses generations with pump wavelength 2150 nm. In Fig. 10(a), the oscillations of the *δ* ^2^ curves at 2150 nm are much weaker than that at 1550 nm shown in Fig. 8(a). The *δ* ^2^ curve of ideal DDW is still the first one that reaches the minimum value of 1.6 × 10^−3^ at *z* = 1.85 mm, similarly to that pumped at 1550 nm. In Fig. 10(b), the ratio *L*_NL_/*L*_D_ of DDW is still the smallest one as in Fig. 8(b). Similarly, the *δ* ^2^ curves in Fig. 10(c) are lower and flatter than those in Fig. 8(c). Figures 10(c,d) show that neither TPA nor FCA loss significantly affects the pulse evolution, which confirms again that the nonlinear losses are negligible in the propagation at 2150 nm.

From Figs.  7–10, the input Gaussian pulses evolve into parabolic pulses. The varying parameters and the nonlinear losses of the waveguide will affect the pulse evolutions. Specifically, the parabolic pulses generated with the pump wavelength at 2150 nm have higher quality than that at 1550 nm. The improved pulse quality is due to the significantly lower nonlinear loss at 2150 nm. We observe that even without loss, the *δ* ^2^ curves at 2150 nm is better than that of corresponding model at 1550 nm. We believe this is due to the lower nonlinearity at 2150 nm than 1550 nm. We note that lower nonlinearity reduces the oscillation and leads to more moderate evolution processes, but requires a longer propagation distance for the formation of parabolic pulse. On the other hand, larger nonlinearity requires a shorter propagation distance for the input pulse to evolve into parabolic pulse but at the expense of larger oscillations as shown in Figs. 8(c)and 10(c).

**Figure 11:**
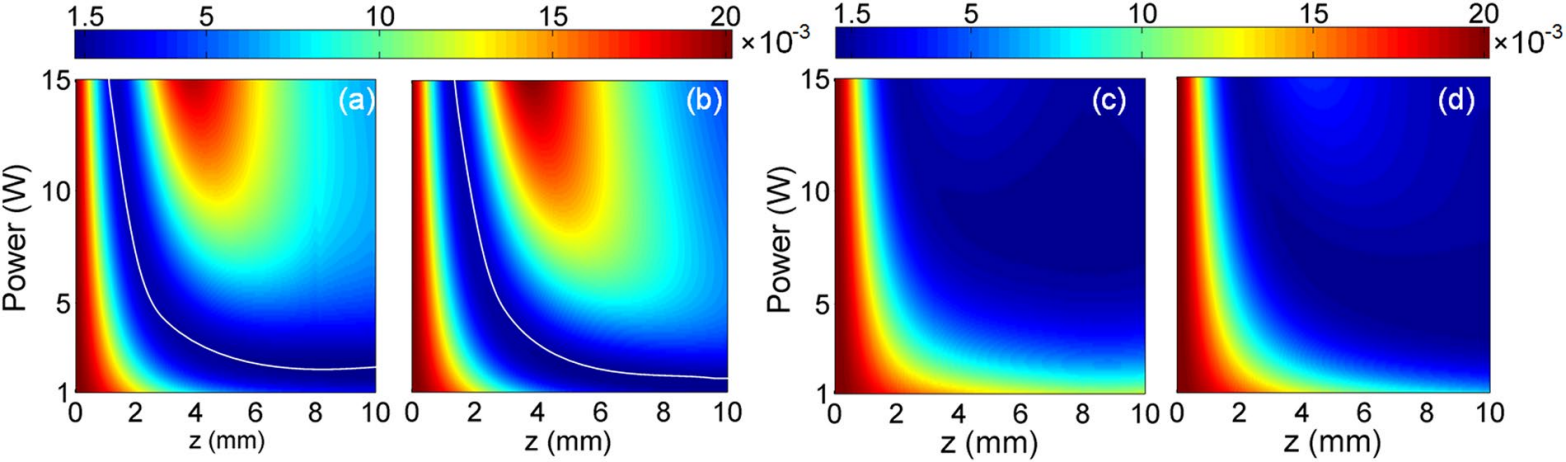
The mismatch *δ* ^2^ for (**a**) Full NIW at 1550 nm, (**b**) Full DDW at 1550 nm, (**c**) Full NIW at 2150 nm and (**d**) Full DDW at 2150 nm. The curves in white in (**a**) and (**b**) represent the minimum *δ* ^2^ at each power *P*.

Figure 11 plots the mismatch *δ* ^2^ as a function of the taper length and input power. The input peak power is varied from 1 to 15 W and the propagation distance remains 10 mm. The white solid curves in Figs. 11(a,b) indicate the minimum *δ* ^2^ at each value of *P*. The minimum *δ* ^2^ values on the two white curves are 1.19 × 10^−3^ and 1.27 × 10^−3^ for Full NIW and Full DDW, respectively. When the pump wavelength is changed to 2150 nm, as shown in Figs. 11(c,d), the values of *δ* ^2^ are much lower than those with 1550 nm pump. The minimum values in Figs. 11(c,d) are 1.03 × 10^−3^ and 9.17 × 10^−4^, respectively. From Fig. 11, high quality parabolic pulses demonstrated in Figs. 9 and 10 should can also be obtained at 1550 nm if the peak power of the input Gaussian pulse is reduced to ~2 W.

## Conclusions

In conclusion, we have investigated the generation of parabolic pulses in tapered a-Si:H-PhWs and a novel scheme for parabolic pulse generation in nonlinearity increasing waveguides is presented. Self-similar theory based on equivalent gain in both nonlinearity increasing and dispersion decreasing schemes is used to design the taper profile. A generalized nonlinear Schrödinger equation, which includes the effect of higher-order dispersions, self-steepening, linear loss, TPA loss, free carrier absorption and dispersion, is used to model optical pulse propagation in the tapered a-Si:H-PhWs. By using numerical simulation, we showed that high quality parabolic pulses can be generated. An input Gaussian pulse will asymptotically evolve into a parabolic pulse in the waveguide tapers. Higher-order effects such as higher-order dispersions and self-steepening do not significantly affect the parabolic pulse generation. Parameters that vary with taper length, either the varying *β*_2_ in NIWs or varying *γ* in DDWs, will deviate the pulse propagation from the ideal cases, but will not significantly degrade the parabolic pulse quality. The lossy terms including the linear loss and TPA will lower the power and the quality of the output pulse significantly at 1550 nm in telecom band. The loss induced pulse degradation is greatly mitigated when the pump wavelength is changed to 2150 nm in mid-IR band. The free carrier absorption in both 1550 nm and 2150 nm are negligible in the propagation. We also found that the pulse quality can be improved by controlling the pulse power to reduce the impact of nonlinearity. The results in this paper will guide on-chip generation and application of parabolic pulses at telecom and mid-infrared wavelengths.

## Methods

### Self-similar theory of parabolic pulses in NIWs

The generation of parabolic similaritons in NIWs is based on the observation that the nonlinear Schrödinger equation (NLSE) with uniform gain and nonlinearity can be transformed into an equation with an increasing nonlinearity but without gain. As a result, the asymptotic parabolic similaritons derived by Fermann *et al*. in active fibers^12^ can also be found in passive NIWs. The propagation of optical pulses in ideal NIWs is described by

7 $$i\frac{\partial A}{\partial z}-\frac{{\beta }_{2}}{2}\frac{\partial {A}^{2}}{\partial {t}^{2}}+{\gamma }_{0}\varepsilon (z){|A|}^{2}A=0,$$

where *A*(*z*, *t*) is the slowly varying envelope of the pulse, *z* is the distance along waveguides and *t* is time. *γ*_0_ is the nonlinear coefficient at *z* = 0. *ε*(*z*) with *ε*(0) = 1 describes the variation of nonlinearity versus *z*. *β*_2_ > 0, is the GVD parameter and considered as a constant. With the definition $u(z,t)=A(z,t)\sqrt{\varepsilon (z)}$, Eq. (7) can be transformed to

8 $$i\frac{\partial u}{\partial z}-\frac{{\beta }_{2}}{2}\frac{{\partial }^{2}u}{\partial {t}^{2}}+{\gamma }_{0}{|u|}^{2}u=i\frac{a(z)}{2}u,\,\,\,\,\,\,\,\,\,\,\,\,\,\,\,\,\,\,\,\,a(z)=\frac{1}{\varepsilon (z)}\frac{d\varepsilon (z)}{dz}.$$

Thus the equivalent gain *a*(*z*) > 0 with  increasing nonlinearity. As a result, pulse propagation in NIWs is equivalent to the propagation in an optical amplifier, and the equivalent gain coefficient is determined by the profile of the nonlinearity variation. Specifically, when the taper profile is

9 $$\varepsilon (z)=\exp ({a}_{0}z),$$

the equivalent gain coefficient becomes a constant. The NLSE of Eq. (8) with normal dispersion and constant gain can be solved analytically by using symmetry reduction. The solutions obtained in this way represent exact self-similar solutions which appear in the asymptotic limit *z* → ∞^49, 50^. The asymptotic solution in NIW with an exponentially increasing nonlinearity profile is given by^12^

10 $$A(z\to {\rm{\infty }},t)\to \{\begin{array}{c}\sqrt{{P}_{0}(z)}{\{1-{[t/{t}_{0}(z)]}^{2}\}}^{1/2}\exp [i\phi (z,t)],\,\,\,\,\,\,\,\,|t|\le {t}_{0}(z),\,\,\,\\ \,\,\,\,\,\,\,\,\,\,\,\,\,\,\,\,\,\,\,\,\,\,\,\,\,\,\,\,\,\,\,\,\,\,\,\,\,\,\,\,\,0,\,\,\,\,\,\,\,\,\,\,\,\,\,\,\,\,\,\,\,\,\,\,\,\,\,\,\,\,\,\,\,\,\,\,\,\,\,\,\,\,\,\,\,\,\,\,\,\,\,\,\,\,\,\,\,\,\,\,\,|t| > {t}_{0}(z).\end{array}$$

In the asymptotic regime, the pulse propagates self-similarly, and maintains its parabolic shape with the exponential scaling of the peak power *P*_0_(*z*) and pulse width *t*_0_(*z*) as

11 $${P}_{0}(z)=\frac{{E}_{0}^{2/3}}{4}{[\frac{2{a}_{0}^{2}}{{\gamma }_{0}{\beta }_{2}}\exp (-{a}_{0}z)]}^{1/3},$$

12 $${t}_{0}(z)=3{E}_{0}^{1/3}{[\frac{{\gamma }_{0}{\beta }_{2}}{2{a}_{0}^{2}}\exp ({a}_{0}z)]}^{1/3},$$

where *E*_0_ is the initial energy of incident pulse and *E*_0_ = 4*P*_0_(*z*)*t*_0_(*z*)/3 for parabolic pulses. Equations (11) and (12) imply that it is only the energy of the initial pulse, regardless of its specific shape, that determines the power and pulse width of the asymptotic parabolic similaritons. The quadratic phase in Eq. (10) is given by

13 $$\begin{array}{c}\phi (z,t)={\phi }_{0}-\frac{{a}_{0}}{6{\beta }_{2}}{t}^{2}+{\gamma }_{0}{\int }_{0}^{z}{P}_{0}(z\text{'})dz\text{'}\\ \,\,\,\,\,\,\,\,\,\,\,\,\,\,\,\,\,\,\,\,\,\,\,\,\,\,\,\,={\phi }_{0}-\frac{{a}_{0}}{6{\beta }_{2}}{t}^{2}-\frac{3{\gamma }_{0}}{{a}_{0}}\,\,\exp (-{a}_{0}z/3)+\frac{3}{4}{(\frac{2{E}_{0}^{2}{a}_{0}^{5}{\gamma }_{0}^{2}}{{\beta }_{2}})}^{1/3},\end{array}$$

where ${\phi }_{0}$ is the initial phase of input pulses. The corresponding chirp is

14 $$\delta \omega (t)=-\partial \phi (z,t)/\partial t={a}_{0}t/3{\beta }_{2}.$$

The chirp is proportional to *a*_0_.

### The self-similar theory of parabolic pulses in DDWs

Parabolic similaritons can also be found in DDWs with appropriate dispersion profile^26^. Optical pulse propagating in DDWs is described by

15 $$i\frac{\partial A}{\partial z}-\frac{{\beta }_{20}}{2}\theta (z)\frac{{\partial }^{2}A}{\partial {t}^{2}}+\gamma {|A|}^{2}A=0,$$

where *β*_20_ is the initial GVD value at *z* = 0. In order to transform Eq. (15) into an equation similar to Eq. (7), we introduce a new coordinate parameter $\kappa ={\int }_{0}^{z}\theta (z\text{'})dz\text{'}$. Thus, Eq. (15) becomes

16 $$i\frac{\partial A}{\partial \kappa }-\frac{{\beta }_{20}}{2}\frac{{\partial }^{2}A}{\partial {t}^{2}}+\frac{\gamma }{\theta (\kappa )}{|A|}^{2}A=0.$$

The new amplitude is defined as

17 $$u(\kappa ,t)=A(\kappa ,t){\theta }^{-1/2}(\kappa ).$$

Substitution of Eq. (17) into Eq. (16) gives

18 $$i\frac{{\rm{\partial }}u}{{\rm{\partial }}\kappa }-\frac{{\beta }_{20}}{2}\frac{{{\rm{\partial }}}^{2}u}{{\rm{\partial }}{t}^{2}}+\gamma {|u|}^{2}u=i(-\frac{1}{2\theta }\frac{d\theta }{d\kappa })u,$$

where *θ*(*z*) is given by

19 $$\theta (z)=\frac{1}{1+{b}_{0}z},$$

The power *P*_0_(*z*) and pulse width *t*_0_(*z*) with hyperbolic gain profile in the normal dispersion region with constant nonlinearity are given by

20 $${P}_{0}(z)=\frac{{E}_{0}^{2/3}}{4}{[\frac{2{b}_{0}^{2}}{\gamma {\beta }_{20}(1+{b}_{0}z)}]}^{1/3},$$

21 $${t}_{0}(z)=3{E}_{0}^{1/3}{[\frac{\gamma {\beta }_{20}(1+{b}_{0}z)}{2{b}_{0}^{2}}]}^{1/3}.$$

The phase is

22 $$\begin{array}{ccc}\phi (z,t) & = & {\phi }_{0}-\frac{{b}_{0}}{6{\beta }_{20}}{t}^{2}+\gamma {\int }_{0}^{z}P(z\text{'})dz\text{'}\\  & = & {\phi }_{0}-\frac{{b}_{0}}{6{\beta }_{20}}{t}^{2}+\frac{{b}_{0}{E}_{0}^{2/3}}{12}{(\frac{2{b}_{0}^{2}{\gamma }^{2}}{{\beta }_{20}})}^{1/3}[1-{(\frac{1}{1+{b}_{0}z})}^{4/3}],\end{array}$$

where *φ*_0_ is the initial phase of the input pulses. The corresponding linear chirp is given by

23 $$\delta \omega (t)=-{\rm{\partial }}\phi (z,t)/{\rm{\partial }}t={b}_{0}t/3{\beta }_{20}.$$

From Eqs. (14) and (23), the parabolic similaritons generated in NIWs and DDWs have similar linear chirp profile. However, because of the different gain coefficients, the evolutions of the power and pulse width of the same initial pulse will be different in the two waveguides.
